# The Crystal Hotel: A Microfluidic Approach to Biomimetic Crystallization

**DOI:** 10.1002/adma.201503931

**Published:** 2015-10-19

**Authors:** Xiuqing Gong, Yun-Wei Wang, Johannes Ihli, Yi-Yeoun Kim, Shunbo Li, Richard Walshaw, Li Chen, Fiona C Meldrum

**Affiliations:** School of Chemistry, University of LeedsLeeds, LS2 9JT, UK; School of Earth and Environment and Leeds Electron Microscopy and Spectroscopy Centre, University of LeedsLeeds, LS2 9JT, UK; Institute of Microwaves & Photonics, School of Electronic & Electrical Engineering, University of LeedsLeeds, LS2 9JT, UK

**Keywords:** bioinspired, biomineralization, calcium carbonate, microfluidics, microstructures, patterning

Control over crystallization to yield particles with defined sizes, morphologies, polymorphs, and orientations is a subject that attracts huge interest, finding applications in the production of pharmaceuticals and biomaterials, and the fabrication of ceramics, nanomaterials, and optical components. While our common expectation is that crystals should exhibit regular, geometric forms that reflect the underlying structure of the crystal lattice, there are of course numerous examples that contradict this picture. One of the best is provided by biominerals such as bones, teeth, and seashells, whose complex morphologies, hierarchical structures, and superior mechanical properties go far beyond those of their synthetic counterparts.^1^

The past few decades have seen significant progress in identifying biomineralization control strategies and employing these synthetically.^2^ Soluble organic molecules and inorganic ions have been widely used to control crystal nucleation and growth^1^,^3^ and to form composite structures,^4^ while organized, insoluble organic matrices such as self-assembled monolayers (SAMs)^5^ and Langmuir monolayers^6^ can direct crystal orientations. Crystallization has been achieved via amorphous precursor phases rather than by ion-by-ion growth, leading to single crystals with unusual morphologies,^7^,^8^ while microemulsion^9^ and liquid crystal^10^ systems can be used to direct the formation of particle assemblies. A number of studies have also applied combinations of strategies, creating self-assembled microlens arrays at the air/water interface,^11^ large micropatterned single crystals via amorphous precursors,^12^ and achieving orientational control on a substrate while controlling morphology using soluble additives.^13^

Few studies, however, have combined such strategies with one of the most fundamental mechanisms by which organisms achieve such remarkable control over biomineralization—confinement. By defining the local environment in which crystallization occurs, organisms can obtain a degree of control that could never be achieved in bulk solution, defining morphologies,^8^,^14^ stabilizing metastable polymorphs,^15^ and achieving temporal control over the supersaturation and additive concentrations. In this article, we profit from advances in microfabrication techniques to create unique, localized crystallization environments in which we can employ multiple strategies to control crystallization. Exhibiting features including confinement, flow, and spatial organization, our “crystal hotel” microfluidic device provides an excellent mimic of biomineralizing systems, in which—thanks to its optical transparency—we can watch how individual crystals grow within confined volumes. Using calcium carbonate as a suitable model system, we use this device to simultaneously apply multiple strategies to control crystallization, thereby generating large crystals with predefined crystallographic orientations, microstructures, and shapes. This system is therefore quite distinct from previous experiments in which continuous-flow microfluidic devices were used to evaluate the effects of biomacromolecules on CaCO_3_ precipitation,^16^ or in which CaCO_3_ and calcium phosphate were precipitated within water/oil (W/O) droplets,^17^ and W/O/W double emulsions respectively.^18^

“Crystal hotel” microfluidic devices were designed to provide a series of confined reaction volumes of well-defined shape, size, and internal patterning into which a controlled flow of reactant ions and additives could be achieved (**Figure** **1** and Figure S1, Supporting Information). These were prepared from poly(dimethylsiloxane) (PDMS) using common lithographic methods^19^ and bonded to a glass slide by plasma treatment. Each hotel comprises a series of eight rooms (R1–R8), which are 120 μm in diameter and 3 μm in height, giving individual volumes of 23.3 pL. The rooms are also internally patterned with an array of PDMS pillars that are 3 μm in diameter and height and set 3 μm apart (Figure 1a–c). This internal patterning provides the opportunity to monitor the formation of crystals with complex morphologies, while the presence of multiple rooms within a single device enables multiple experiments to be performed simultaneously. There is an approximately sevenfold drop in the gas pressure from R1 (closest to the inlet) to R8 (Figure 1d,e), where this was calculated by measuring the arc length of a liquid in the feeding structure when applying a constant gas pressure (Figure S2, Supporting Information). The supersaturation therefore builds fastest in R1, and crystallization is observed in R1 before R8.

**Figure 1:**
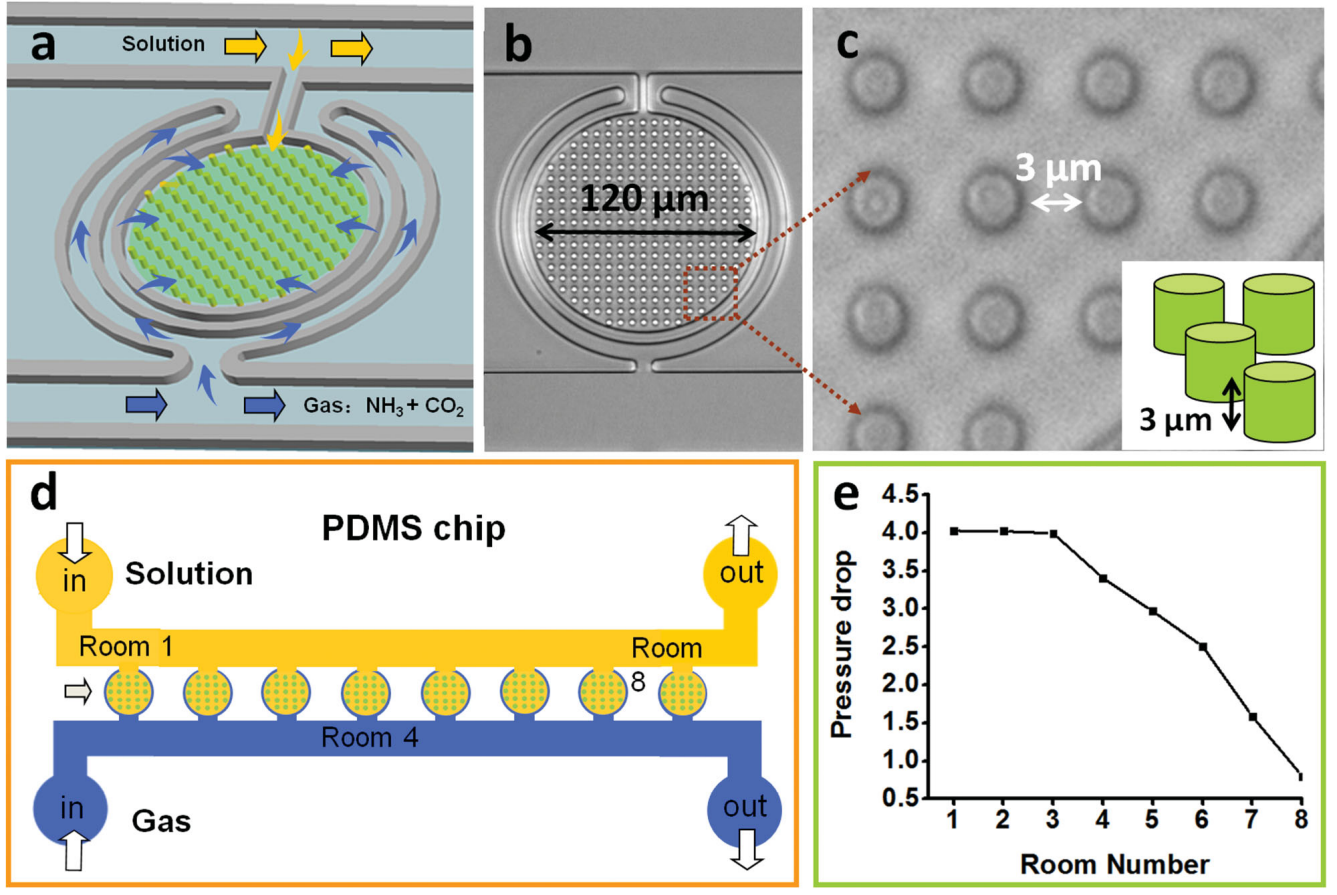
Schematic diagram of the Crystal hotel. a–c) The PDMS device is composed of circular rooms, numbered R1–R8, which are internally patterned with pillars. Each room is surrounded by a circular channel (blue arrow) from which it is separated by a PDMS membrane, which is used to provide gas flow to the room, and a direct feeding channel that supplies solution (yellow arrow). d) The device is positioned on an inverted microscope and crystallization is achieved by supplying CaCl_2_ solution and vapor released from (NH_4_)_2_CO_3_ powder via two syringe pumps. e) A sevenfold pressure drop in the gas feed occurs across the eight rooms.

Calcium carbonate precipitation in the rooms is achieved by delivering NH_3_ and CO_2_ gases (arising from the decomposition of (NH_4_)_2_CO_3_ powder) through one feed channel, and a solution of CaCl_2_, either on its own or with MgCl_2_ and poly(acrylic acid) (PAA) as additives, through a second independent feed channel. The solution feed channel is connected directly to each chamber, whereas the channel carrying the NH_3_ and CO_2_ gases encircles each chamber and permeates through a thin layer of PDMS. This design ensures continuous mineralization, and that this is localized within the hotel rooms. In a standard crystallization experiment, the hotel rooms are first filled with the CaCl_2_ solution at 10 μL min^−1^, and after establishing a steady CaCl_2_ solution flow, the flow rate is reduced to 1 μL h^−1^. The supply of the NH_3_(g) and CO_2_(g) into the device is then initiated, inducing CaCO_3_ precipitation.^20^

Crystallization was first investigated in the absence of additives using “standard” growth conditions of [CaCl_2_] = 5 × 10^−3^ m, solution flow rate = 1 μL h^−1^, and gas flow rate = 10 μL min^−1^. The development of the crystals in the individual hotel rooms was imaged using optical microscopy, and the data presented is representative of at least 32 rooms. Precipitation was first observed after ≈30 min in R1 and appeared as a background of poorly-defined particles (**Figure** **2**a; Figure S3, Supporting Information). These were isotropic under polarization microscopy, where this is consistent with the production of amorphous calcium carbonate (ACC). These precursor particles dissolved over time and were replaced by a single, crystalline particle of size 2–3 μm after ≈1.5 h (Figure 2a); by comparison, it took 2 h to generate a comparable crystal in R8 (Figure 2b). The polymorphs of the crystals generated were identified using Raman microscopy after removing the PDMS template, and were calcite in all cases, as shown by the bands at 1087 and 712 cm^−1^ (*ν*_1_ and *ν*_4_ bands), and 281 and 153 cm^−1^ (lattice modes) (Figure 2c).

**Figure 2:**
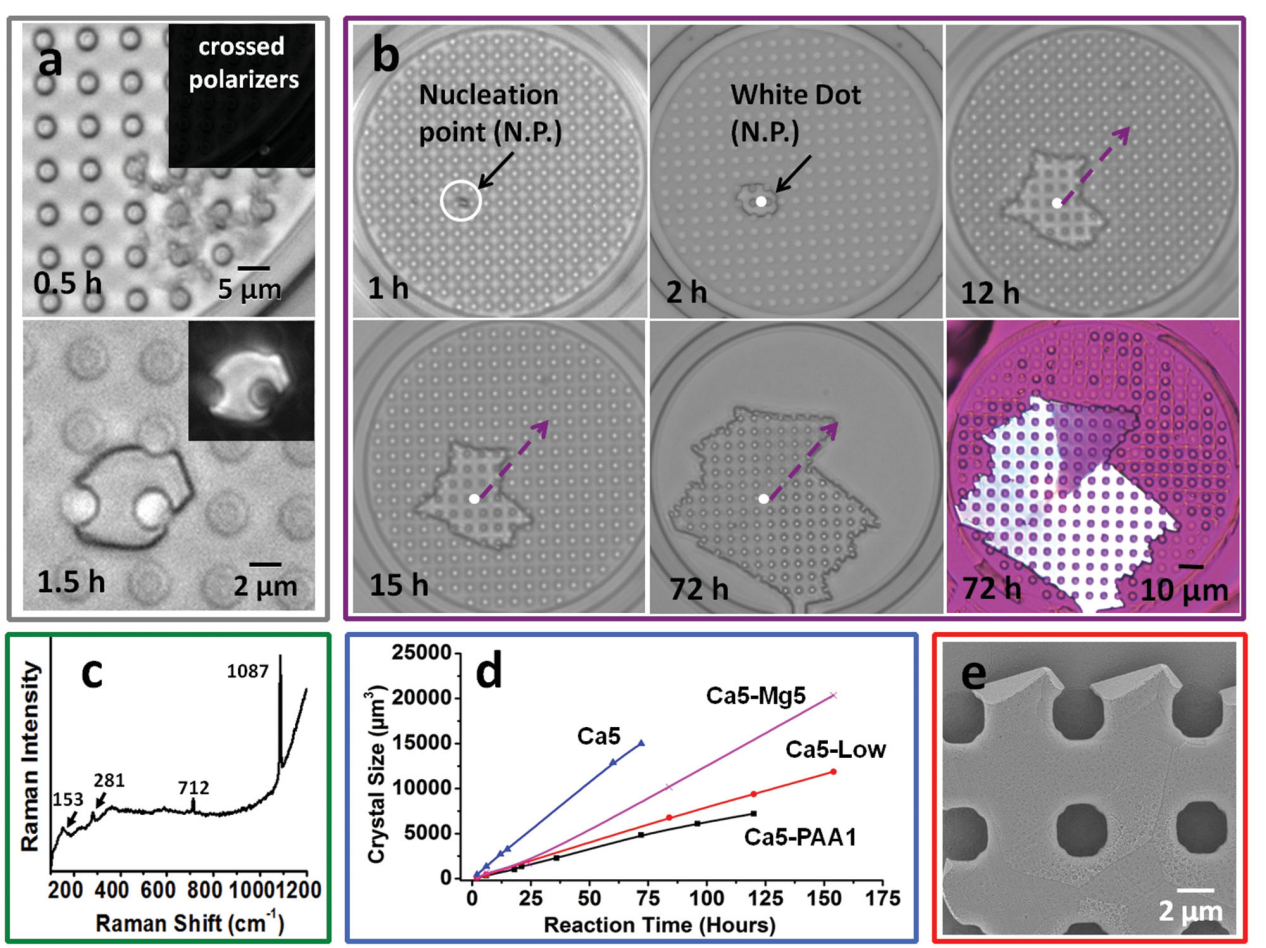
a–c) Calcite crystals precipitated in the crystal hotel in the absence of additives under growth conditions of [CaCl_2_] = 5 × 10^−3^ m, gas flow rate 10 μL min^−1^ and solution flow rate 1 μL h^−1^. a) Diffuse particles are present after 30 min, which are consistent with ACC; these are replaced by a single crystal after 1.5 h. The inset shows an image recorded between crossed polars. b) The growth of an individual crystal over 72 h. Growth is pinned such that it is retarded along the direction indicated, where this results in a domain of different orientation, as observed using a quarter-wave plate. c) Raman spectrum of a CaCO_3_ crystal showing that it is calcite. d) Graph of the change in the crystal area with time. PAA, Mg, and Ca5 all correspond to the standard conditions given for (a–c), with [PAA] = 1 μg mL^−1^ and [MgCl_2_] = 5 × 10^−3^ m. Low gas flow rate is identical to Ca5-low, with the exception of a gas flow rate of 1 μL min^−1^. e) The growth front of a crystal, showing rhombohedral faces characteristic of calcite.

The configuration of this device provides a unique opportunity to study the morphological development of crystals grown within a template of complex geometry. Even at the early stages of formation, crystals grew to meet the pillars such that their morphologies were defined by the template (Figure 2a). This demonstrates that ion flow to the growing crystal is not prevented by the presence of the pillars. The full growth sequence of a crystal can be seen in Figure 2b, where measurement of the area of the crystal with time reveals an extremely constant growth rate (Figure 2d, Ca5). The crystal always grew to be entirely space-filling, reaching sizes of ≈10 μm after 2 h, ≈30 μm after 15 h, and ≈50 μm after 60 h, and no evidence of preferential nucleation sites within the rooms was obtained. In the example shown in Figure 2b, the crystal exhibits some planar faces even at small (5 μm) sizes, where these become more evident with growth. The growth front of the templated crystals also shows well-defined rhombohedral facets, which are consistent with the crystallography of calcite (Figure 2e). The faceting seen at the interface with the pillar simply reflects the shape of the original pillar. The irregular morphology of the crystal shown in Figure 2b is likely due to a defect/impurity site, which initially pins crystal growth along the direction indicated between 12 and 15 h, with growth resuming after this time. Notably, this pinning site gives rise to the formation of a secondary grain with a crystallographic orientation dissimilar to the parent crystal, such that the product crystal contains two single crystal domains.

The influence of the growth conditions on crystallization was also investigated, and showed that the flow rate and CaCl_2_ concentration could be used to control the number of crystals formed in each room. At the fixed liquid flow rate of 1 μL h^−1^, a decreased gas flow rate from 10 μL min^−1^ (Figure 2d, Ca5) to 1 μL min^−1^ (Figure 2d Ca5-Low) resulted in an approximately 50% decrease in growth rate, but little change in the crystal morphologies. The gas flow rate can also be used to control the number of crystals produced in each room such that two to three crystals are typically formed at a flow rate of 10 μL min^−1^, as compared with only one at 1 μL min^−1^ (Figure S4, Supporting Information). This provides us with a fine control over the number of crystals produced in each room. Higher calcium concentrations of [CaCl_2_] = 10 × 10^−3^ m also increased the number of crystals in each room from an average of one at [CaCl_2_] = 5 × 10^−3^ m to two to three at [CaCl_2_] = 10 × 10^−3^ m at the same flow rate (Figure S5, Supporting Information).

Soluble additives (Mg^2+^ and PAA) were then also explored as a means to further refine the structures of the crystals precipitated within the hotel rooms, where this is a key strategy used in biomineralization.^1^ Experiments were performed by combining the additives with the CaCl_2_ solution. **Figure** **3**a shows the growth sequence of a calcite crystal from a solution of [CaCl_2_] = 5 × 10^−3^ m and [MgCl_2_] = 5 × 10^−3^ m under standard flow conditions. As compared with the additive-free experiments, crystal growth is ≈40% slower in the presence of Mg^2+^, such that crystals are 10 μm in size at 18 h as compared with more than 30 μm in size in the additive-free solutions; this is consistent with the known inhibitory effect of Mg^2+^ on calcite growth.^21^ The Mg^2+^ ions also had a significant effect on the crystal morphologies in that pinning events were rare. Crystals thus formed with isotropic morphologies, as exemplified by the crystal shown in Figure 3a. Crystals formed under these conditions were characterized using energy-dispersive X-ray analysis (EDXA), which showed a Mg content of approximately 5 mol% (Figure S6a, Supporting Information), and Raman microscopy confirmed they were calcite (Figure S6c, Supporting Information).

**Figure 3:**
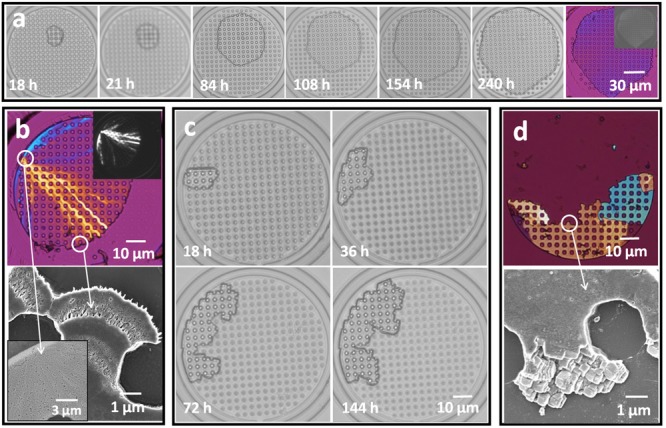
Calcite crystals precipitated in the crystal hotel in the presence of additives. a) Growth sequence of crystals precipitated at [CaCl_2_] = 5 × 10^−3^ m, [MgCl_2_]:[CaCl_2_] = 1:1, solution flow rate 1 μL h^−1^, and gas flow rate 10 μL min^−1^, where the final image is recorded using a quarter wave plate. b) Crystals precipitated under identical conditions but with [MgCl_2_]:[CaCl_2_] = 3:1, where the polarized image and the growth front suggest a polycrystalline structure. c) Growth sequence of a crystal precipitated at [CaCl_2_] = 5 × 10^−3^ m, [PAA] = 1 μg mL^−1^, and the standard flow rates given above. d) A polarized image and the growth front of a crystal precipitated under the conditions given in (c).

Increase in the magnesium concentration to [MgCl_2_]/[CaCl_2_] = 3:1 led to more rounded morphologies, and a Mg content of approximately 18 mol% (Figure 3b and Figure S6b, Supporting Information). However, this was accompanied by a loss in the single-crystal structure as shown by the polarized light image, which shows radial features emanating from the nucleation center (Figure 3b and Figure S7a, Supporting Information). The growth front of these crystals also comprised elongated crystallites rather than the rhombohedra seen in the absence of Mg^2+^ (Figure 2e), and Raman microscopy confirmed the calcite polymorph (Figure S6c, Supporting Information). Looking then to PAA, a concentration of 1 μg mL^−1^ gave slightly slower growth than achieved with [MgCl_2_]/[CaCl_2_] = 1:1 (Figure 2d), and a representative crystal and its growth sequence is shown in Figure 3c. Crystals precipitated in the presence of PAA were characteristically highly irregular in shape, due to pinning in multiple positions. The crystals were also typically oligocrystalline in structure, which is again likely to derive from pinning of the growth front, and showed a growth front comprising multiple co-oriented rhombohedra (Figure 3d and Figure S7b, Supporting Information). Further examples of crystals precipitated under different conditions are given in Figure S8 (Supporting Information).

While selection of the reagent concentrations and flow rates, and the room size, shape, and internal structuring can deliver control over features including the number of crystals per room, their morphologies, and single crystal/polycrystalline structures, it did not deliver one of the key features of many biominerals—orientation. This additional target was achieved by functionalizing the glass substrate with a SAM terminating in carboxylic acid groups in situ within the Crystal Hotel (Figure S9, Supporting Information).^22^

Crystals were precipitated on the bare glass and functionalized substrates under the standard flow conditions with [CaCl_2_] = 5 × 10^−3^ m. The SAMs supported more rapid growth of crystals than the unfunctionalized substrates, where this is consistent with the ionotropic properties of the charged surface. The orientations of the crystals were investigated using polarization microscopy and electron backscatter diffraction (EBSD). **Figure** **4**a,b shows cross-polarized optical images of crystals nucleated in three hotel rooms on an unfunctionalized glass substrate and carboxylic acid terminated SAM. The different colors of the crystals in R1–R3 in Figure 4a show that they nucleate on the unfunctionalized substrate with a wide range of orientations, while the crystals deposited on the carboxylic acid terminated SAM present homogenous color maps both across and within rooms, which demonstrate preferred orientation (Figure 4b).

**Figure 4:**
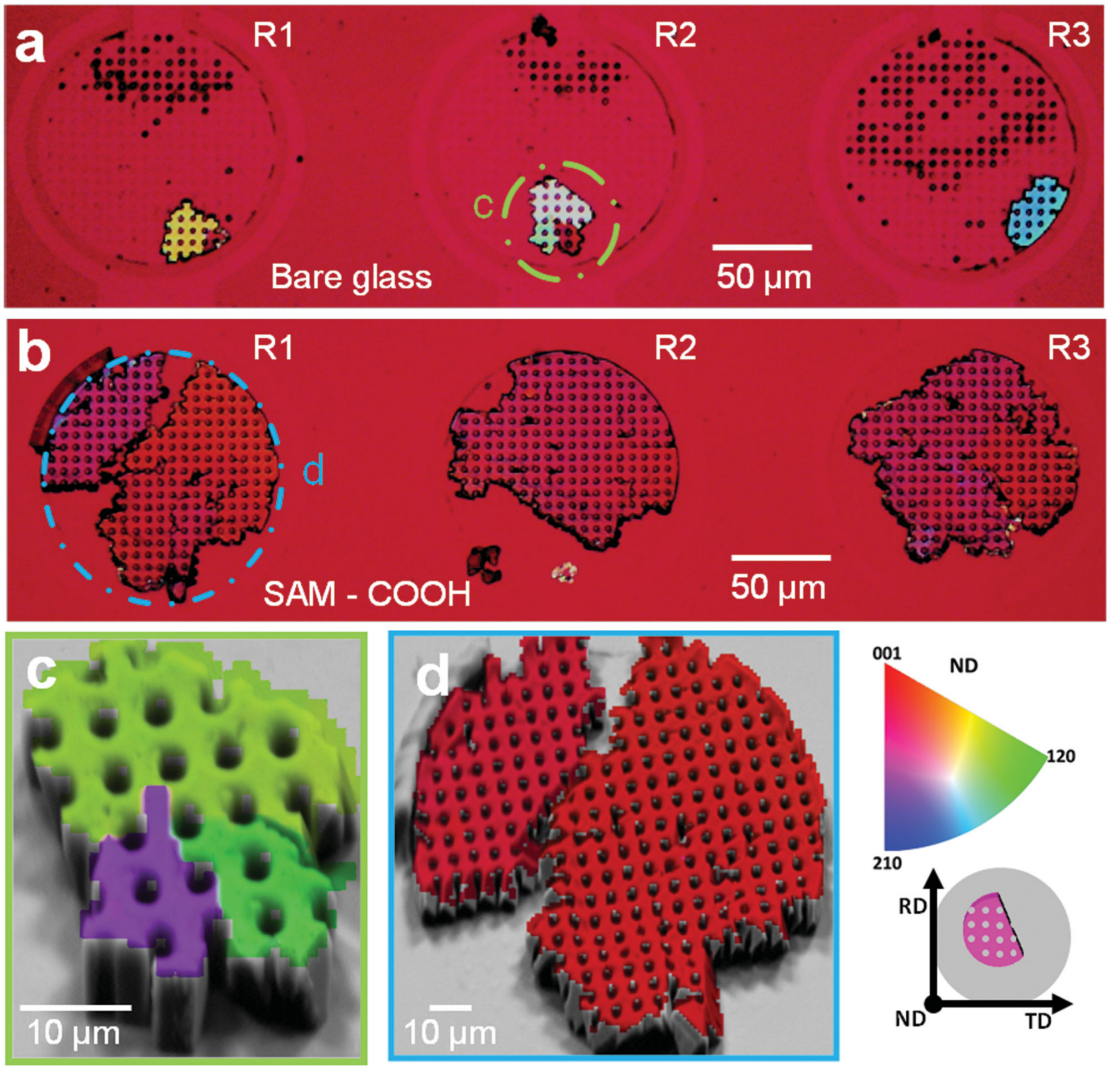
Calcite crystals precipitated at [CaCl_2_] = 5 × 10^−3^ m, solution flow rate 1 μL h^−1^, and gas flow rate 10 μL min^−1^. a,b) Optical images recorded under crossed polarizers of crystals nucleated on a) an unfunctionalized glass substrate and b) a carboxylate terminated SAM. c,d) Electron image projected EBSD maps collected from the circled rooms shown in (a) and (b). The substrate normal component (ND) is shown together with the corresponding inverse pole figure map (right), where RD is the rolling direction and TD the transverse direction.

EBSD was then used to determine the crystal orientations. EBSD maps (Figure 4c,d) and the corresponding pole figures (Figure S10, Supporting Information) of the crystals shown in Figure 4a,b confirmed that the crystals nucleated on the bare glass had no preferential orientation, while those nucleated on the SAMs were preferentially aligned with their *c*-axes nearly normal to the substrate, as is consistent with published data.^23^ These results were further confirmed by recording a series of images of crystals grown on both types of substrates under a rotating cross polarizer (Figure S11 and Movie S1 and S2, Supporting Information).

These results highlight the unique level of control over crystallization—or more generally materials synthesis—that could be obtained using microfluidic devices. Many of the individual features of our system can be achieved using alternative strategies. Thus, templates such as colloidal crystals have been used to generate polycrystalline materials with reverse-opal structures,^24^ and reaction conditions have been controlled to template the formation of single crystals with complex forms.^25^ A notable example by Aizenberg et al. filled a pillared template with ACC before triggering transformation to a large patterned single crystal of calcite.^11^ Achieving both templated single crystal formation and oriented crystal growth is more challenging, but has been obtained by homo or heteroepitaxial growth on inorganic substrates.^26^ With the ability to provide a continuous and controlled flow of reagents into the reaction chamber, the microfluidic approach described here has a demonstrated potential for an additional level of control over these existing systems. The additional opportunity to watch crystals growing in situ within the template also enables the device design and reaction conditions to be readily optimized for the production of a target material.

In summary, this work demonstrates that the controlled environments provided by microfluidic devices can be used to achieve levels of control over crystallization comparable to those in biomineralization processes. Application of a combination of biogenic strategies including constrained physical environments, tailored reaction conditions, and surface functionalities are sufficient to generate single crystals with predefined macroscopic shapes, patterned microstructures, and crystallographic orientations. As a successful proof-of-concept, this study employed a simple circular template featuring a 2D pattern of pillars, and focused on the biologically and environmentally important material calcium carbonate. Work is currently underway to extend our approach to more complex 3D structures with nanometer-scale features and technologically important single crystals for applications in areas such as optical devices.

## Experimental Section

“Crystal hotel” microfluidic devices were fabricated from PDMS and were bonded to glass slides. Devices were used immediately after fabrication, both without further functionalization and after functionalization with SAMs. Functionalization with SAMs was carried out by depositing (3-aminopropyl)triethoxysilane monolayers on the interior surfaces of the device and then chemically modifying the end groups with docosanoic acid to give a carboxylated surface.

CaCO_3_ was precipitated within the hotel rooms by delivering the reagents using two syringe pumps, one loaded with CaCl_2_ solution and the other with (NH_4_)_2_CO_3_ powder, which releases NH_3_(g)+CO_2_(g) gas on decomposition. The reactions were allowed to continue for up to 240 h. Experiments were also performed where MgCl_2_ or PAA was added to the CaCl_2_ solution. Crystal morphologies were determined in situ in the crystal hotel devices using polarized light microscopy and scanning electron microscopy after removing the PDMS to expose the CaCO_3_ crystals adhered to the glass substrate. Crystal polymorphs were determined by Raman microscopy. EBSD was performed to investigate the orientation of the calcite crystals on the glass substrates, where both pole figures and EBSD maps were generated.

## Supporting Information

As a service to our authors and readers, this journal provides supporting information supplied by the authors. Such materials are peer reviewed and may be re-organized for online delivery, but are not copy-edited or typeset. Technical support issues arising from supporting information (other than missing files) should be addressed to the authors.
