# Supplementary material for: The Crystal Hotel: A Microfluidic Approach to Biomimetic Crystallization
Source: Adv Mater. 2015 Oct 19;27(45):7395–400. doi: 10.1002/adma.201503931 (PMC4722853; doi:10.1002/adma.201503931)
Supplement: Supplementary file 1 — Supplementary [file adma0027-7395-sd1.pdf]

Copyright WILEY-VCH Verlag GmbH & Co. KGaA, 69469 Weinheim, Germany, 2015.

ADVANCED MATERIALS

Supporting Information

for *Adv. Mater.,* DOI: 10.1002/adma.201503931

The Crystal Hotel: A Microfluidic Approach to Biomimetic Crystallization

*Xiuqing Gong, Yun-Wei Wang, Johannes Ihli, Yi-Yeoun Kim, Shunbo Li, Richard Walshaw, Li Chen, and Fiona C. Meldrum**

Supporting Information

The Crystal Hotel: A Microfluidic Approach to Biomimetic Crystallization

*Xiuqing Gong, Yun-Wei Wang, Johannes Ihli, Yi-Yeoun Kim, Shunbo Li, Richard Walshaw, Chen Li and Fiona C. Meldrum**

Extended Methods Section

Materials and General Methods: Analytical grade $(NH_4)_2CO_3$, $CaCl_2 \cdot 2H_2O$, $MgCl_2 \cdot 6H_2O$, poly(acrylic acid) (PAA), (3-aminopropyl)triethoxysilane (APES), diisopropylethylamine (DIEA), docosanedioic acid (DCDA), 1-Hydroxybenzotriazole hydrate (HOBt) and (Benzotriazol-1-yloxy) tripyrrolidinophosphonium hexafluorophosphate (PyBOP) were purchased from Sigma-Aldrich and were used as received. Polydimethylsiloxane (PDMS) structures were prepared using a SYLGARD 184 silicon elastomer kit. Aqueous stock solutions were prepared using Milli-Q water, 18.2 MΩ cm^{-1}. Glassware used to prepare solutions was soaked overnight in 10% w/v NaOH, and was then rinsed with dilute HCl, before finally washing with Milli-Q water. Glass slides were placed overnight in Piranha solution (70:30 wt% H_2SO_4: H_2O_2) and were then washed copiously with Milli-Q water before drying with ethanol and nitrogen gas.

Manufacture of Microfluidic Devices: “Crystal hotel” microfluidic devices were fabricated using standard soft lithographic methods. [1] The microfluidic mold was prepared by pouring SU-8 2002 over a 4 inch silicon wafer, and spin-coating at a speed of 1000 rmp for 30 seconds. This was followed by photo mask deposition and UV radiation to develop the template structure. A PDMS precursor mixture (10:1 base to catalyst) was poured over the

prepared lithographically fabricated silicon master and cured in a ventilated oven at 60 °C for 120 min. Inlets and outlets (1 mm in diameter) were punched in the cured PDMS substrate, which was then plasma cleaned 1 min, Harrick Plasma Cleaner RD-002), along with piranha-cleaned glass slides. The Crystal Hotel was then assembled by gently pressing the plasma exposed surfaces (glass and PDMS) together and finally transferring the assembled device to the oven for a further 5 min to strengthen the PDMS-glass bonding. Devices were used immediately after fabrication, both without further functionalization and after functionalization with self-assembled monolayers, as described below.

Functionalization of the Glass Substrate in the Crystal Hotel: The glass slide forming the base of the crystal hotel was also functionalized with a self-assembled monolayer (SAM) to change the surface chemistry and thus direct the orientation of the precipitating crystals. This was carried out by depositing alkoxyaminosilane monolayers on the interior of the device and then chemically modifying the end-groups. In order to achieve this, the chips must be exceptionally clean and the surfaces fully hydroxylated via plasma cleaning. 1% APES was diluted with 95% methanol and 4% acetic acid and then injected into the freshly prepared PDMS chips. The surface reaction was stopped after 20 min by rinsing the chips with methanol 3 times. The end group modification was achieved as follows. In order to generate carboxylate end groups, APES was coupled with docosanedioc acid (DCDA) (APES-DCDA). 0.27 mM of DCDA (1 equiv, MW = 370.6) and 2.8 equivalents of DIEA (132 μL, MW = 129.3) was added to 104 mL of 2:1 chloroform/DMF and stirred for 3 h to dissolve. Then 0.1 equiv of HOBT (MW = 135) and 0.1 equivalent PyBOP (MW = 520) were added as coupling agents. The carboxylate activation reaction was allowed to proceed for 30 min prior to injecting into the PDMS chips. After soaking overnight in the coupling solution, the chips were rinsed twice with 1:1 chloroform/DMF, twice with a 1:1 chloroform/ methanol mixture, and once with methanol. [2]

Crystallization within Crystal Hotel Devices: Calcium carbonate was precipitated within the confines of the hotel rooms by delivering the reagents ($CaCl_2$ solution and $NH_{3(g)}$ + $CO_{2(g)}$ gasresulting from the decomposition of $(NH_4)_2CO_{3(s)}$) to the device using two Harvard Apparatus PHD 2000 syringe pumps, one loaded with $CaCl_2$ solution and the other with $(NH_4)_2CO_3$ powder (Figure 1a). The device was initially flushed with $CaCl_2$ solution (5 mM) through the first feed channel for 5 min at a flow rate 10 μL min^{-1} to remove any impurities in the channels, after which time the flow rate was reduced to 1 μL hr^{-1}. The system was then left undisturbed for 10 min to establish a stable liquid flow. After this, the gas ($NH_{3(g)}$ + $CO_{2(g)}$) was pumped through the second feed channel at a rate of 1-10 μL min^{-1}. The $NH_{3(g)}$ is of particular importance as it raises the solution pH, thus facilitating the precipitation of $CaCO_3$.[3] The reactions were allowed to continue for 16-264 h, after which time pumping was discontinued and the delivery tubes removed. Experiments were also performed where additives were added to the 5 mM calcium solution to concentrations of 5 mM $MgCl_2$ or 1 μg mL^{-1} poly(acrylic acid) (PAA).

The use of multiple reaction chambers placed successively in a single design was chosen as it provides us with the opportunity to study $CaCO_3$ nucleation and crystal growth under a decreasing range of supersaturation across the separate chambers in a single experiment. This is achievable as the gas ($NH_{3(g)}$ and $CO_{2(g)}$) feeding pressure drops (Δp) with increasing channel length as given by Equation 1.

$$\Delta p = \frac{f \rho L {\mu_m}^2}{2 D_h} \qquad (1)$$

Where L is the length of channel, D_h is the dimension of channels, μ_m is the mean fluid velocity, ρ is the fluid density and f the Darcy friction factor. [4] In the current case we have a distance of 200 µm between adjacent chambers (center to center). This results in a ~7 fold drop in gas pressure from R1 (closest to the inlet) to R8, Figure 1e. The pressure drop was calculated by measuring the arc length of a liquid in the feeding structure when applying a constant gas pressure (Figure S1). The existing pressure drop directly governs the velocity of gas ($NH_{3(g)}$ and $CO_{2(g)}$) permeation though the thin PDMS layer to each reaction chamber as is evident from Equation 2. Here (P) is permeability factor of PDMS for a specific gas, p_2 is the gas feeding pressure, p_1 is the gas permeate pressure, l is the PDMS membrane thickness and N is the steady-state gas flux through the PDMS "membrane". [5]

$$N = \frac{P(p_2 - p_1)}{l} \tag{2}$$

Now assuming that the gas delivery to the chambers consist of 3 sequential steps. The rapid saturation of the gas phase with $CO_{2(g)}$ and $NH_{3(g)}$ in the syringe, subsequent transportation of $CO_{2(g)}$ and $NH_{3(g)}$ across the PDMS membrane, and lastly the diffusion of "CO_2 and NH_3" across the gas-liquid interface into the solution, with the intermediate step being the slowest given the diffusion coefficients of ammonia and carbon dioxide in water are 1.64×10^{-5} cm^2 s^{-1} and 1.92×10^{-5} cm^2 s^{-1}, [4] while 7.08×10^{-10} cm^2 s^{-1} and 2.1×10^{-10} cm^2 s^{-1} in PDMS respectively we can study crystallization under different supersaturation regimes by simply adjusting the gas feeding pressure (by varying the gas flow rate) while maintaining the "Ca^{2+}" liquid flow rate, here set to 1 µL h^{-1}.

Characterization: Crystal morphologies were determined *in situ* in the crystal hotel devices using polarized light microscopy using a Nicon Eclipse LV100 equipped with a circular rotating specimen stage to facilitate orientation studies and using scanning electron

microscopy (SEM). Samples for SEM were prepared by immersing the microfluidic devices in ethanol for 3 h to weaken the PDMS to glass bonding, before peeling the PDMS from the glass. This procedure leaves the $CaCO_3$ crystals adhered to the glass substrate. The glass substrate was then rinsed with ethanol to remove residual PDMS and reagent solutions before drying in air. The dried glass was mounted on an SEM stub using adhesive conducting pads, and images were acquired using a FEI Nova NanoSEM 650 after gold coating. Crystal polymorphs were determined by Raman microscopy, using a Renishaw 2000 inVia-Raman microscope equipped with a 785 nm diode laser. Electron backscatter diffraction (EBSD) was also performed to determine the orientation of the calcite crystals on the glass substrate. The crystals were examined using an **FEI Quanta 650** environmental SEM with a Centaurus EBSD system. As the crystals grew on a planar substrate, no polishing step was required prior to imaging. Samples after PDMS removal were coated with 10 nm of carbon. EBSD maps were obtained at 15 kV, with an imaging step size of 1 μm^2 and a total acquisition time of 20 min for a fixed area of 60 μm^2. The AZtecHKL software package of Oxford instruments was used to create pole figures and EBSD maps.

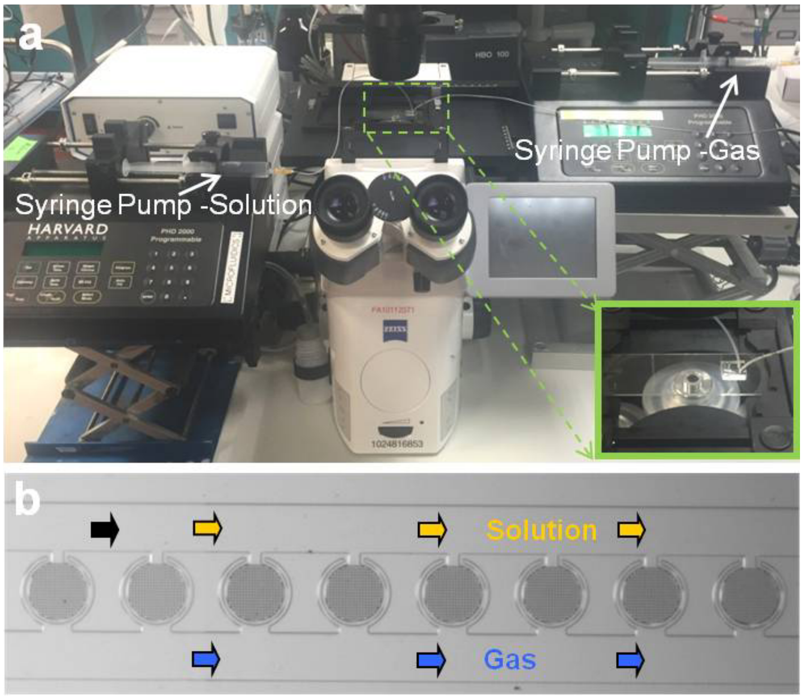

Figure S1. a) Calcium carbonate was precipitated within the hotel rooms by delivering the reagents using two syringe pumps (Harvard Apparatus PHD 2000), one loaded with $CaCl_2$ solution and the other with $(NH_4)_2CO_3$ powder which releases $NH_{3(g)}$ + $CO_{2(g)}$ gas on decomposition. b) Transparent "Crystal hotel" devices were fabricated from PDMS (polydimethylsiloxane), were bonded to glass slides and were studied under light microscopy.

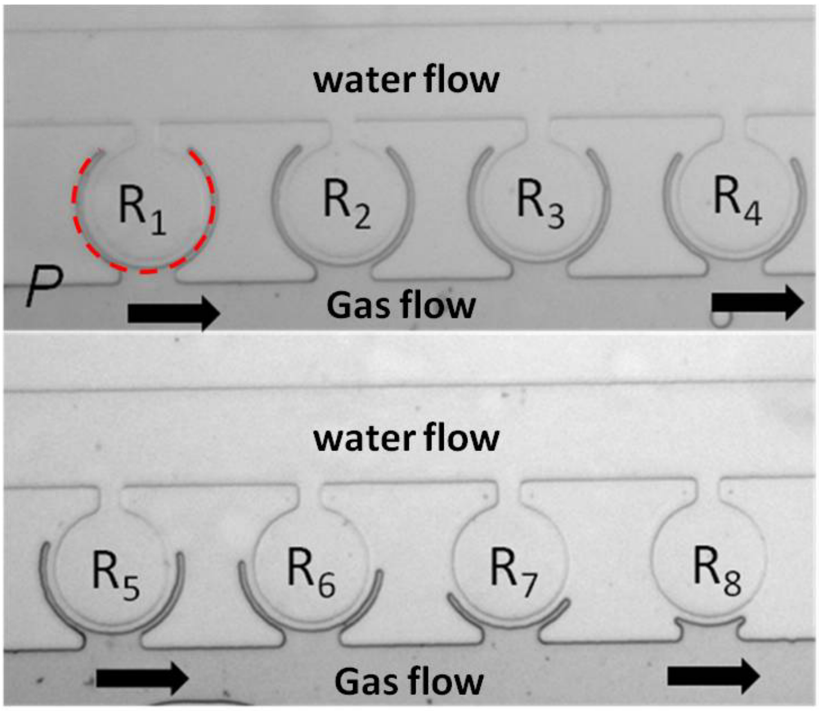

Figure S2. Illustration of the gas feeding pressure drop with increasing channel length and chamber number (R1-R8). The arrow indicates the gas flow direction. The relative feeding pressure drop is calculated by measuring the arc length (dashed red arc) across the subsequent chambers. To do this, we firstly filled all the channels with water and then pumped gas at flow rate of 10 μL min^{-1} to squeeze the water out of the chip. The gas will also squeeze the water in the arc area due to the compressibility and permeability of PDMS.

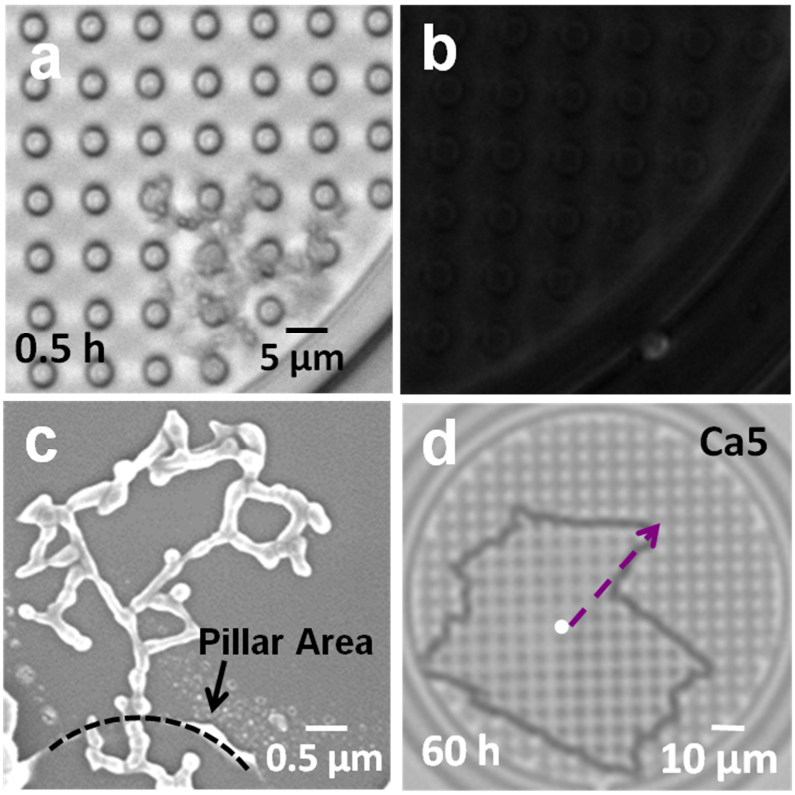

Figure S3. Calcite crystals precipitated in the crystal hotel in the absence of additives under growth conditions of $[CaCl_2] = 5 \times 10^{-3}$ M, gas flow rate 10 µL min^{-1} and solution flow rate 1 µL h^{-1}. (a) Diffuse particles are present after 30 min which are consistent with amorphous calcium carbonate (ACC); b) shows an image recorded between crossed polarized light; c) shows a corresponding SEM image and d) The growth of an individual calcite crystal precipitated in the crystal hotel after 60 hour in the absence of additives under growth conditions of $[CaCl_2] = 5 \times 10^{-3}$ M, gas flow rate 10 µL min^{-1} and solution flow rate 1µL h^{-1}.

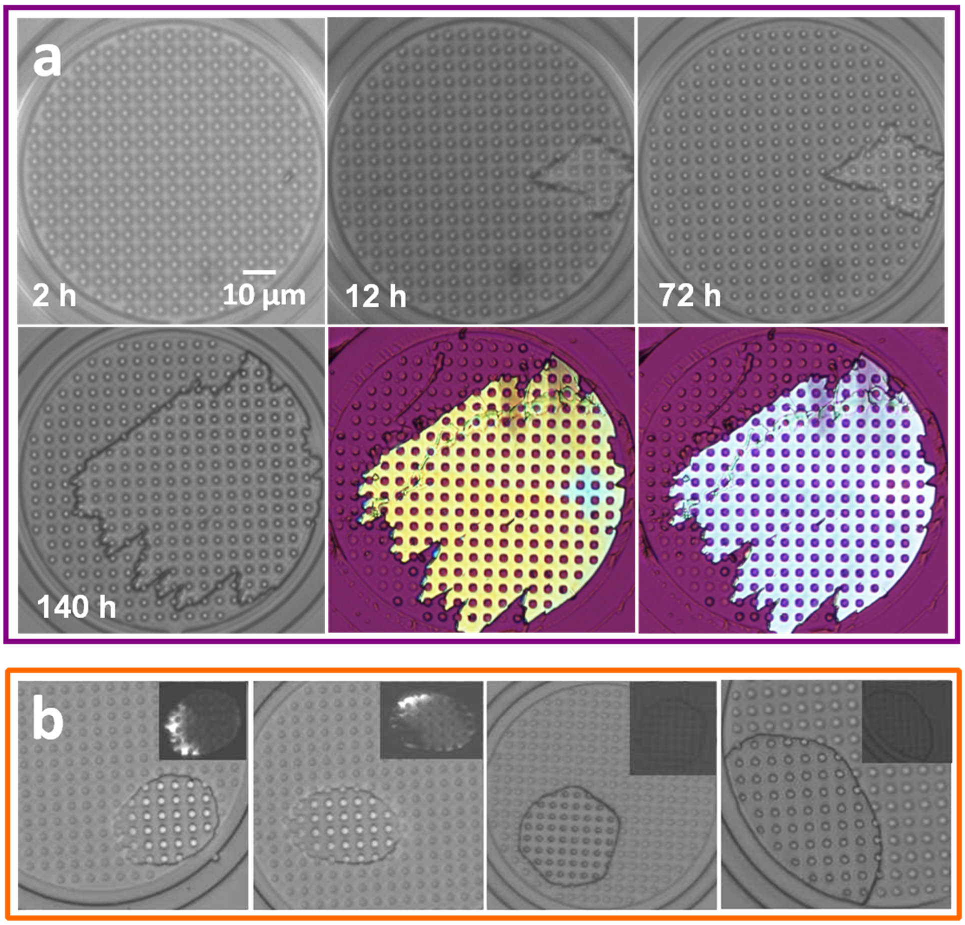

Figure S4. Crystal growth of calcite crystal in a) a hotel room observed by optical microscopy with time and b) Growth of calcite crystals in different hotel rooms, where [$CaCl_2$] = 5 × 10^{-3} M, the gas flow rate is 1 μL min^{-1} and the solution flow rate 1 μL h^{-1}.

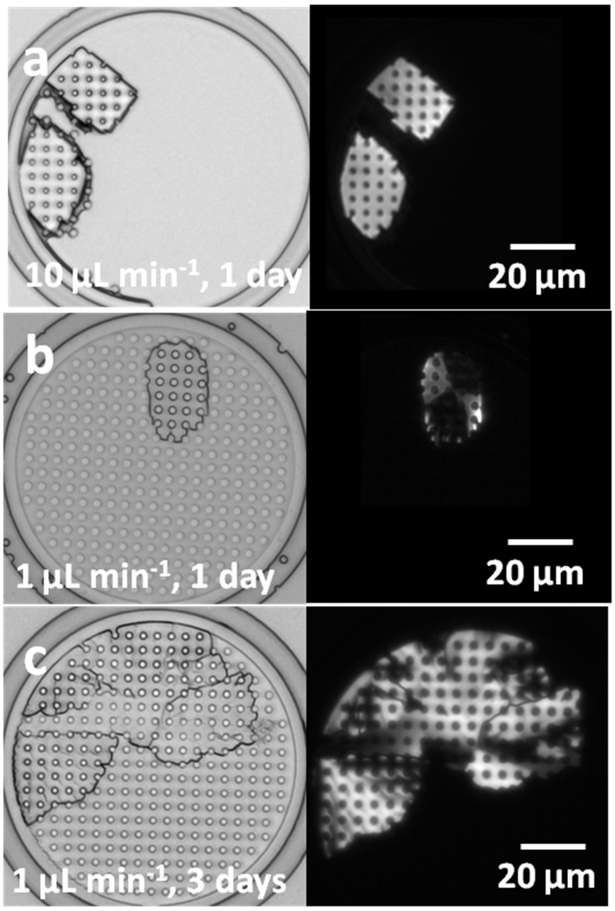

Figure S5. Crystal growth of calcite crystal in a reaction chamber observed by optical microscopy at concentration of [$CaCl_2$] = 10 × 10^{-3} M with the liquid feeding rate is 1 μL h^{-1} and the gas flow rate is a) 10 μL min^{-1} after 1 day (the pillars are not seen as the room as the room is filled with static liquid), b) 1 μL min^{-1} after 1 day and c) 1 μL min^{-1} after 3 days.

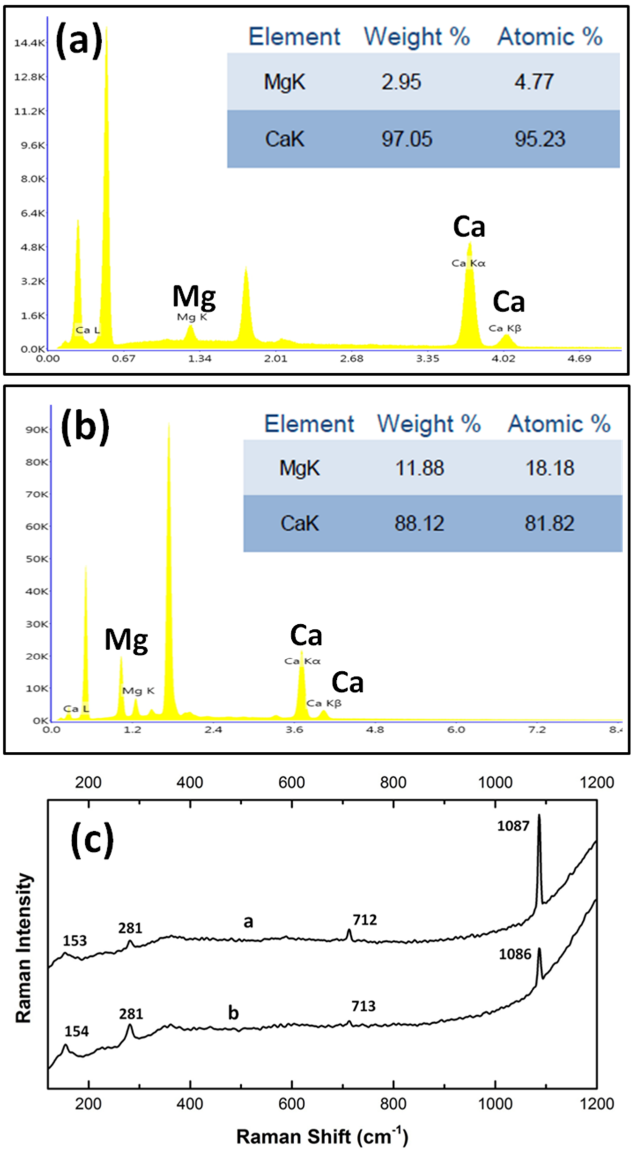

Figure S6. EDX spectra of calcite crystals precipitated in the crystal hotel in the presence of $MgCl_2$, with at [$CaCl_2$] = 5 × 10^{-3} M, solution flow rate 1 μL h^{-1} and gas flow rate 10 μL min^{-1}. (a) [Mg]:[Ca] = 1:1, (b) [$MgCl_2$]:[$CaCl_2$] = 3:1 and c) corresponding Raman microscopy.

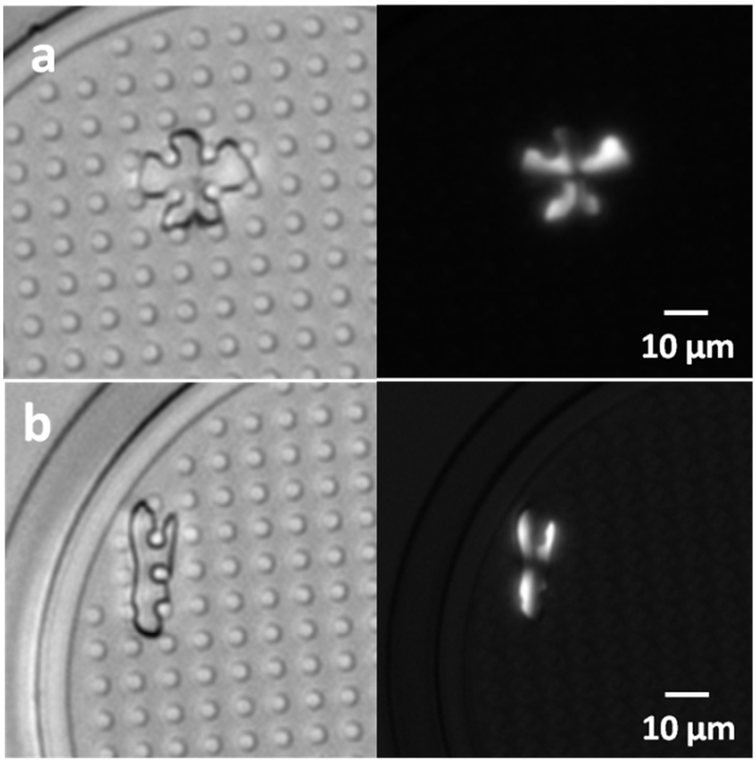

Figure S7. Crystal growth crystal in a reaction chamber observed by optical microscopy after 3 h. The liquid solution is a) [$MgCl_2$]/[$CaCl_2$] = 3:1 and b) PAA = 1 µg mL^{-1}.

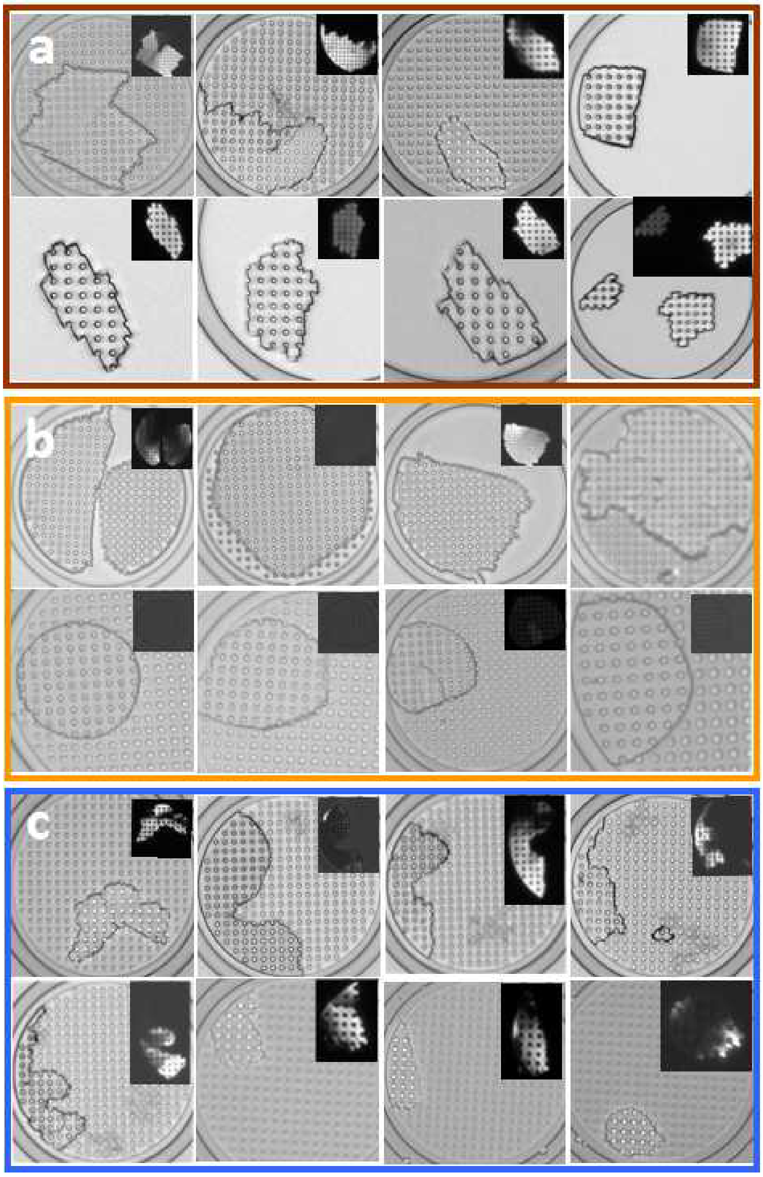

Figure S8. Calcite crystals precipitated in the crystal hotel under growth conditions of $[CaCl_2] = 5 \times 10^{-3}$ M, gas flow rate 1 μL min^{-1} and solution flow rate 1 μL h^{-1} (a) in the absence of additives, (b) in the presence of $[MgCl_2] = 5 \times 10^{-3}$ M and (c) in the presence of 1 μg mL^{-1} PAA.

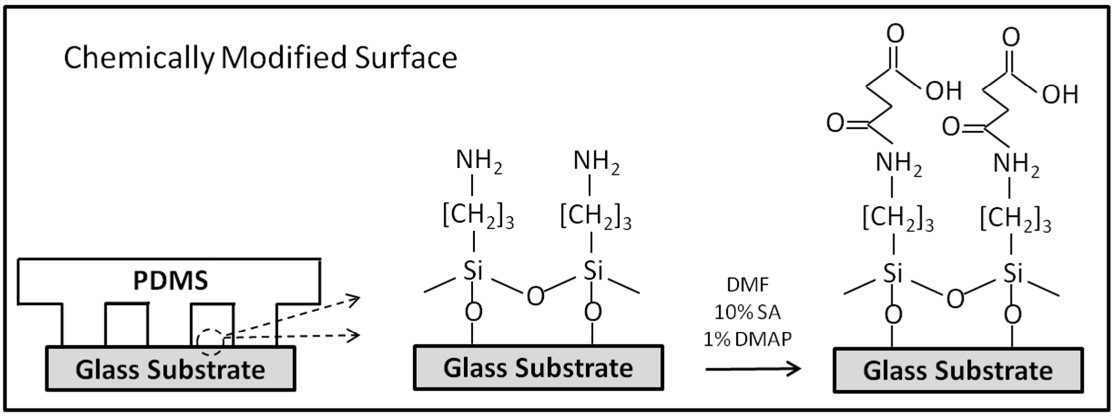

Figure S9. Illustration of the surface functionalization (-COOH) procedure. Linkage chemistries used in coupling of acidic functional groups to aminosilane SAMs.

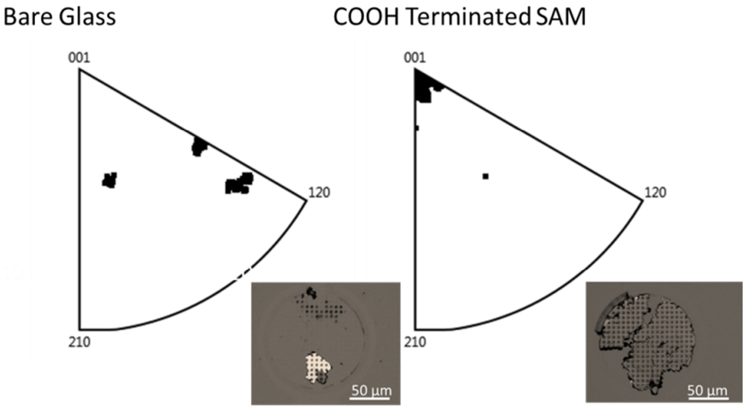

Figure S10. Inverse pole figures generated from EBSD data of the crystals shown in Figure 4, where these were nucleated in single hotel rooms on either an unfunctionalized glass substrate or a carboxylate terminated SAM. These data show the orientation of crystals normal to the substrate.

a **Bare Glass**

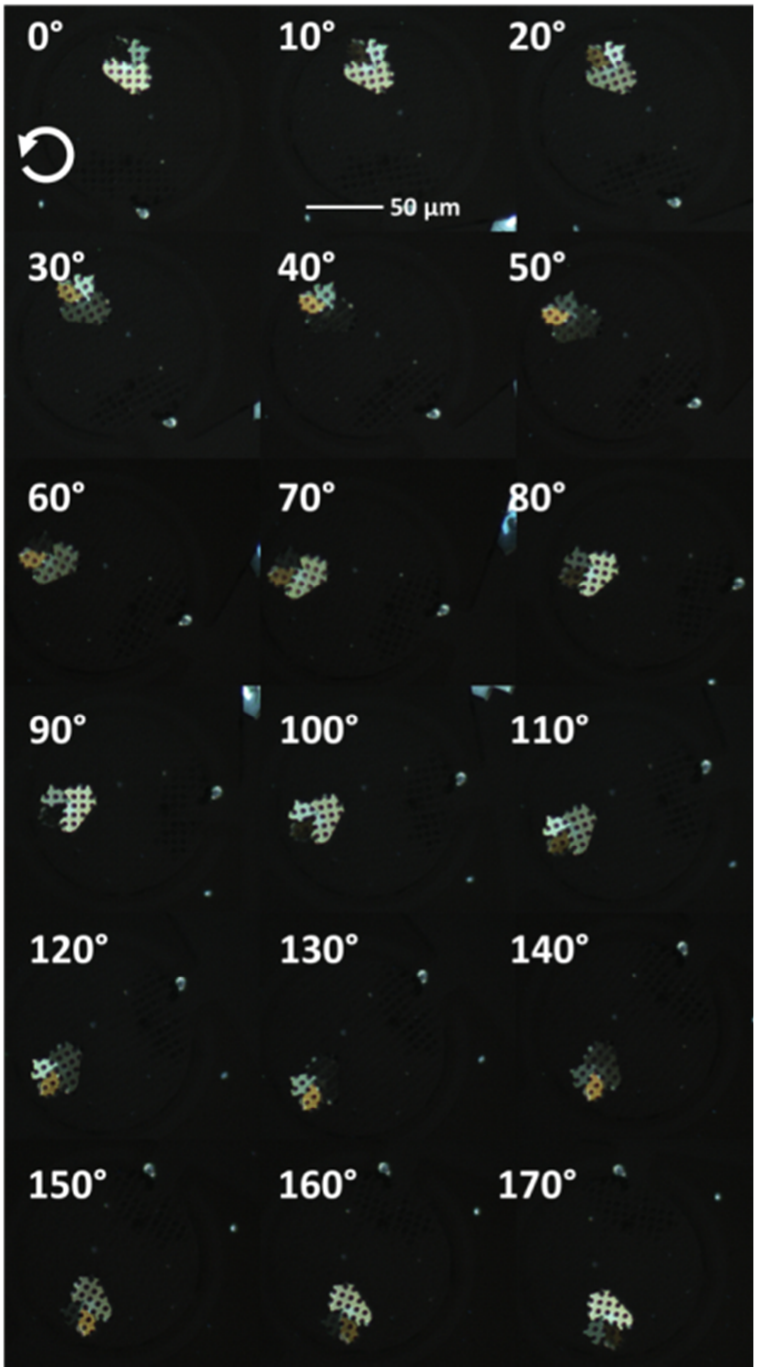

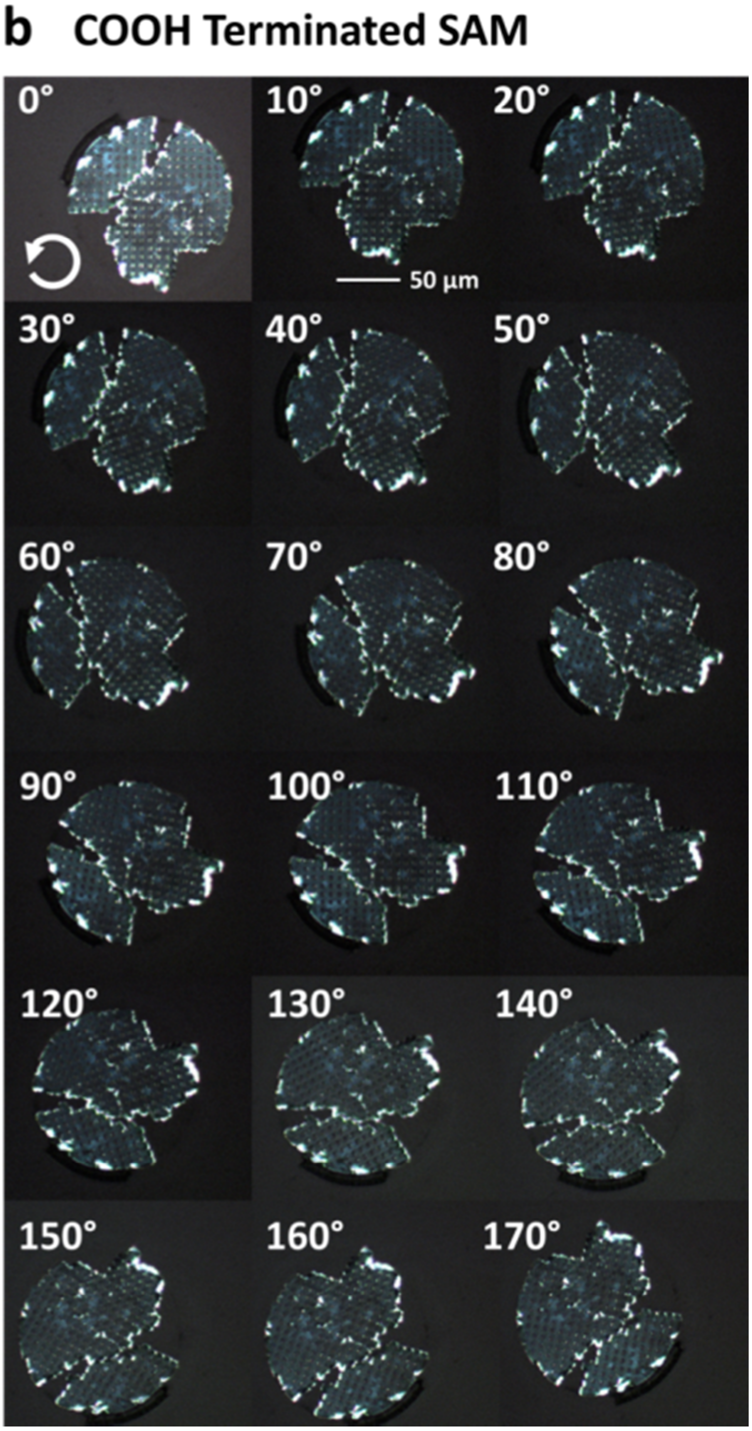

Figure S11. Series of optical micrographs of crystals (a) nucleated on an unfunctionalized glass surface and (b) on a carboxylate terminated SAM. Images were recorded under crossed polarizer with the sample stage rotating 10° per frame. The crystals are grown from a of 5 × 10^{-3} M $CaCl_2$ solution supplied at a rate of 1 μL h^{-1} with a gas flow rate of 1 μL min^{-1}.

Supporting Movies S1 and S2: Shown are two series of optical micrographs collected of crystals nucleated on an unfunctionalized glass surface and on a carboxylate terminated SAM.

References

[1] X. Gong, A. V. Patil, A. P. Ivanov, Q. Kong, T. Gibb, F. Dogan, A. J. deMello, J. B. Edel, *Anal. Chem.* **2014**, *86*, 835.

[2] D. D. Archibald, S. B. Qadri, B. P. Gaber, *Langmuir* **1996**, *12*, 538.

[3] J. Ihli, P. Bots, A. Kulak, L. G. Benning, F. C. Meldrum, *Adv. Funct. Mater.* **2013**, *23*, 1965.

[4] I. Papautsky, T. Ameel, A. B. Frazier, presented at *ASME, Proceedings of Int. Mech. Eng Congress Expos Proc (IMECE)*, **2001**.

[5] T. Merkel, V. Bondar, K. Nagai, B. Freeman, I. Pinnau, *J. Polym. Sci. Part B Polym. Phys* **2000**, *38*, 415.
